# Assessment of Video Capsule Endoscopy in the Management of Acute Gastrointestinal Bleeding During the COVID-19 Pandemic

**DOI:** 10.1001/jamanetworkopen.2021.18796

**Published:** 2021-07-30

**Authors:** Shahrad Hakimian, Daniel Raines, George Reed, Mark Hanscom, Lilia Stefaniwsky, Matthew Petersile, Prashanth Rau, Anne Foley, David Cave

**Affiliations:** 1 Department of Medicine, Division of Gastroenterology, University of Massachusetts Medical School, Worcester; 2 now with Department of Medicine, Division of Digestive Diseases, University of California, Los Angeles; 3 Section of Gastroenterology, Louisiana State University Health Sciences Center, New Orleans

## Abstract

**Importance:**

Evaluation of acute gastrointestinal (GI) bleeding using invasive endoscopic procedures comprising the standard of care (SOC)—upper endoscopy and colonoscopy—can expose the endoscopy staff to SARS-CoV-2. Video capsule endoscopy (VCE) does not generate aerosols and only requires 1 person to manage the procedure.

**Objective:**

To examine the safety of VCE for the initial evaluation of GI bleeding at the peak of the COVID-19 pandemic to identify signs of active bleeding while minimizing patient and personnel exposure, saving personal protective equipment, and avoiding invasive or unnecessary procedures.

**Design, Setting, and Participants:**

A multicenter (UMass Memorial Medical Center and Louisiana State University Health Sciences Center) retrospective cohort study including 146 patients with COVID-19 who received VCE as the first-line diagnostic modality was conducted from March 15 to June 15, 2020, compared with SOC in January 2020 for evaluation of GI bleeding. The association between treatment and outcomes was estimated using multivariable regression adjusting for potential confounders. Propensity score matching was used to verify the results.

**Main Outcomes and Measures:**

The primary end point was detection of active bleeding or stigmata of recent bleeding. Secondary end points included the number of patients requiring any invasive procedures, number of additional procedures, rates of rebleeding and rehospitalization, transfusion requirements, and mortality.

**Results:**

Among 146 patients, 92 (63.0%) were men; mean (SD) age was 64.93 (14.13) years in the COVID-19 group and 61.33 (13.39) years in the SOC group. Active bleeding or stigmata of recent bleeding was observed in 44 (59.5%) patients in the COVID-19 group compared with 18 (25.0%) in the SOC group (adjusted odds ratio, 5.23; 95% CI, 2.23 to 12.27). Only 36 patients (48.7%) in the COVID-19 group required any invasive procedure during the hospitalization compared with 70 (97.2%) in the SOC group (adjusted odds ratio, 0.01; 95% CI, 0.001 to 0.08). The mean (SD) number of invasive procedures was 0.59 (0.77) per patient in the COVID-19 group compared with 1.18 (0.48) per patient in the SOC group (adjusted difference, −0.54; 95% CI, −0.77 to −0.31). Both approaches appeared to be safe and there was no significant difference in transfusion requirements, rebleeding, rehospitalization, or in-hospital mortality. No mortality was attributed to GI bleeding in either group.

**Conclusions and Relevance:**

In this cohort study, first-line diagnostic evaluation of acute GI bleeding using VCE appeared to be a safe and useful alternative to the traditional approach of upper endoscopy and colonoscopy. Use of VCE was associated with increased detection of active bleeding and a reduced number of invasive procedures and unnecessary exposure of personnel to SARS-CoV-2 and use of personal protective equipment.

## Key Points

**Question:**

Can video capsule endoscopy be safely used as an alternative to standard endoscopic procedures for the initial evaluation of gastrointestinal bleeding during the COVID-19 pandemic?

**Findings:**

In this cohort study of 146 patients (74 with COVID-19), active bleeding was more frequently identified with video capsule endoscopy as the first strategy (59.5%) compared with conventional endoscopic evaluation (25.0%). The number of invasive procedures was significantly decreased with video capsule endoscopy without increased risk of rebleeding or compromising safety.

**Meaning:**

The results of this study suggest that video capsule endoscopy can serve as a safe alternative to the standard endoscopic evaluation of gastrointestinal bleeding because it reduces the number of invasive procedures, personnel involved, and use of personal protective equipment.

## Teaser

This cohort study examines the use of video capsule endoscopy vs standard invasive diagnostic procedures to evaluate gastrointestinal (GI) bleeding in patients with COVID-19.

## Introduction

SARS-CoV-2, a single-stranded enveloped RNA virus, is responsible for the COVID-19 pandemic.^1^ SARS-CoV-2, found in the gastric, duodenal, and rectal epithelia of infected patients,^2^ can be transmitted via aerosols, droplets, and physical contact.^3,4^ Esophagogastroduodenoscopy (EGD) and colonoscopy, routinely used in the evaluation of gastrointestinal (GI) disorders, are both known to produce aerosols, thus potentially exposing endoscopy personnel to SARS-CoV-2.^4^ However, although many elective GI procedures can be deferred or canceled during surges of the pandemic,^5^ the evaluation of acute GI bleeding is often urgent and as such cannot be deferred. Current guidelines recommend early EGD^6^ and/or colonoscopy^7^ within 24 hours as the first-line diagnostic and therapeutic modalities for acute GI bleeding.

More recently, several studies have suggested a role for video capsule endoscopy (VCE) in the management of acute GI bleeding.^8,9,10^ Video capsule endoscopy is a minimally invasive tool that does not require sedation or GI manipulation and can visualize the upper, middle, and lower GI tract^8^ without generating aerosols. Reports suggest that VCE is more effective than conventional endoscopy in detecting the site of active bleeding in many patients^9^ and hence serves as a valuable tool in guiding further diagnostic and therapeutic interventions if needed. These changes in protocol, prioritizing early VCE as a first-line diagnostic procedure, have been shown to be safe^9,10^ and well tolerated. In this study, we sought to examine the use of VCE for the initial evaluation of suspected GI bleeding in patients with suspected or established COVID-19 as a first-line tool to help minimize patient and personnel exposure to SARS-CoV-2, limit unnecessary contacts, save personal protective equipment (PPE), and avoid invasive and unnecessary procedures.

## Methods

### Design

We designed a multicenter retrospective cohort study aimed at assessing the comparative use of VCE as a triaging tool as an alternative to standard of care (SOC) for evaluation of GI bleeding. The study was conducted at UMass Memorial Medical Center and Louisiana State University Health Sciences Center and approved by the institutional review boards at both institutions. Study personnel (S.H., M.H., L.S., M.P., and A.F.) collected data via review of medical records and completed data entry into a standardized secure online database, Research Electronic Data Capture (REDCap),^11^ which was hosted at UMass Memorial Medical Center. Demographic, clinical, and outcome data were collected and analyzed. The institutional review boards for each facility granted a waiver of informed consent for the patients receiving SOC, because this was retrospective data collection. The patients undergoing VCE were regarded as receiving SOC and were not required to provide consent for the study; rather, only routine informed consent for procedures was required by the institutional review boards. The waiver also covered retrospective data collection for the VCE cohort. This study followed the Strengthening the Reporting of Observational Studies in Epidemiology (STROBE) reporting guideline.

The video capsule group included 74 patients with COVID-19 admitted between March 15 to June 15, 2020, who underwent VCE as the first-line diagnostic modality for evaluation of GI bleeding. A total of 72 historical controls were identified from January 1 to 31, 2020, a period before the known spread of the COVID-19 pandemic in the US; these patients had undergone SOC. Inclusion criteria for patients in both groups required that they be consecutive, hemodynamically stable adults (age >18 years) with suspected GI bleeding as evidenced by the presence of melena, hematochezia, hematemesis, and/or severe anemia. Patients who developed signs and symptoms of GI bleeding during their hospitalization for other indications were also included. Exclusion criteria for VCE included dysphagia, gastroparesis, previous intestinal surgery, abdominal radiotherapy, and the presence of an implantable cardiac device. Patients were followed up for 30 days after their episode of GI bleeding for assessments of rebleeding and mortality.

Invasive procedures included EGD, colonoscopy, enteroscopy, conventional angiography, and surgery. Diagnostic procedures were defined as any of the abovementioned invasive procedures and computed tomographic angiography and VCE, which we defined as minimally invasive. An upper source of bleeding was defined to be within the reach of an EGD, a middle source as a small intestinal bleed beyond the second portion of the duodenum, and a lower source as a colonic source of bleeding. A presumptive source was defined as any lesions or blood typically associated with a bleeding source with or without signs of active bleeding or stigmata of bleeding.

Both centers used the PillCam SB 3 and RAPID software, version 8.00 or higher (both Medtronic). Patients fasted for 6 to 8 hours before capsule ingestion per standard protocol. Patients then swallowed the capsule with 118 to 237 mL of water. No purgative bowel preparation was used. The real-time viewer was used immediately after ingestion to check for blood in the stomach and again within a few hours to check for bleeding in the small intestine. If bleeding was noted, the study was truncated, data were downloaded from the recorder, and a video created on a workstation was reviewed to determine the exact source of bleeding. If no blood was seen on the initial review, the capsule was allowed to record for up to 12 hours before download and review.

The primary end point of the study was detection of active bleeding or stigmata of recent hemorrhage. Secondary end points included the number of patients undergoing invasive procedures, number of additional procedures needed for evaluation, rates of rebleeding and rehospitalization, transfusion requirements, and rates of mortality.

To perform VCE, we assumed 1 staff member, albeit for a brief encounter, aided the patient in swallowing the video capsule and later collected and cleaned the equipment. For EGD, colonoscopy, and enteroscopy, we assumed 4 staff members were present during the procedure, which included the endoscopist, endoscopy nurse, endoscopy technologist, and anesthesia staff. We assumed no trainee involvement and no additional preprocedure and postprocedure staff for this estimate.

### Statistical Analysis

Patient characteristics are described as means (SDs) or as percentages. Continuous variables were analyzed using *t* test and binary variables were analyzed using χ^2^ or Fisher exact tests. Multivariate logistic and linear regression estimated adjusted odds ratios (ORs) or differences of means for primary and secondary outcomes. As a secondary analysis, the primary and secondary outcomes were compared in propensity score–matched patients. Key variables with a standardized difference in absolute value greater than 0.15 were considered for matching. Propensity scores were estimated using a logistic regression model for the COVID-19 vs SOC cohorts that included age, sex, race/ethnicity, Glasgow-Blatchford score, cirrhosis, and kidney failure. One-to-one matching within center using a caliper of 0.15 maximized the sample size (n = 54 per arm) while balancing the key characteristics (eTable 2 in the Supplement). For the primary outcome of active bleeding, secondary analyses included a multivariable-adjusted OR (COVID-19 vs SOC) in the population on common support of the propensity score, and in the matched population adjusted for characteristics with standardized difference greater than 0.15 in absolute values. Sample size was based on data from 2 randomized clinical trials.^8,12^ With 70 patients per group, there would be greater than 80% power for differences in rates of active bleeding detection of 25% or for ORs of 3.0 or greater. The significance threshold was α = .05; all tests were 2 sided where appropriate. Unpaired *t* test, Fisher exact test, logistic regression, and linear regression assumed independent samples. Statistical analysis was conducted with Stata, version 16.1 (StataCorp LLC).

## Results

The study included a total of 146 patients, with 74 patients in the COVID-19 group and 72 patients in the SOC group. Of these, 118 patients were enrolled from UMass Memorial Medical Center and 29 from Louisiana State University. A total of 92 (63.0%) of the patients were men and 54 (37.0%) were women; mean (SD) age was 64.93 (14.13) years in the COVID-19 group and 61.33 (13.39) years in the SOC group; Table 1 includes baseline characteristics that were assessed for potential inclusion in the propensity score (eTable 1 in the Supplement includes the list of all baseline characteristics collected in the overall population). Matching resulted in 54 patients in each group (eTable 2 in the Supplement). The analysis of results for the overall population and the matched population is summarized in Table 2 along with secondary analyses of the primary outcome (eTable 3 in the Supplement). As defined by the study objectives, all 74 patients in the COVID-19 group underwent VCE as the first procedure. In the SOC group, 60 patients (83.3%) underwent EGD, 9 (12.5%) underwent colonoscopy, and 3 (4.2%) underwent VCE as the first procedure (Table 3).

**Table 1.** Baseline Characteristics and Matched Variables in the Overall Population

Characteristic	COVID-19, No. (%) (n = 74)	SOC, No. (%) (n = 72)	Standard difference
Sex			
Female	29 (39.2)	25 (34.7)	0.092
Male	45 (60.8)	47 (65.3)	
Age, mean (SD), y	64.93 (14.13)	61.33 (13.39)	0.261
Race/ethnicity			
White	49 (66.2)	40 (55.6)	0.218
African American	12 (16.2)	16 (22.2)	−0.152
Hispanic	4 (5.4)	6 (8.3)	−0.115
Other^a^	9 (12.2)	10 (13.9)	−0.051
Cirrhosis	13 (17.6)	17 (23.6)	−0.149
Kidney failure	5 (6.8)	4 (5.6)	0.05
Presenting symptom			
Hematemesis	8 (10.8)	11 (15.3)	−0.132
Melena	35 (47.3)	27 (37.5)	0.198
Hematochezia	3 (4.1)	16 (22.2)	−0.555
Anemia	28 (37.8)	18 (25)	0.277
Medications			
Nonsteroidal anti-inflammatory drugs	9 (12.2)	14 (19.4)	−0.199
Aspirin	33 (44.6)	27 (37.5)	0.144
P2Y12 inhibitor	10 (13.5)	8 (11.1)	0.073
Warfarin	14 (18.9)	8 (11.1)	0.218
Direct oral anticoagulant	10 (13.5)	4 (5.6)	0.272
Heparin	2 (2.7)	2 (2.8)	−0.005
No anticoagulant	33 (44.6)	39 (54.2)	−0.191
Glasgow-Blatchford score, mean (SD)^b^	8.43 (2.36)	7.56 (2.90)	0.331

Abbreviation: SOC, standard of care.

^a^ This designation includes combined data for Asian and Native American individuals, data from patients with Middle Eastern race/ethnicity not individually collected, and data from patients of unknown race specified as Other.

^b^ Posssible range on the Glasgow-Blatchford scale is 0 to 23. Scores greater than 6 indicate a patient has a greater than 50% chance of requiring intervention.

**Table 2.** Main Treatment Outcomes and Safety for Overall and Matched Populations

Variable	Overall population				Matched population			
	SOC (n = 72)	COVID-19 (n = 74)	*P* value ^a^	Adjusted OR/difference (95% CI)^b^	SOC (n = 54)	COVID-19 (n = 54)	*P* value^a^	Adjusted OR/difference (95% CI)^b^
Main outcomes								
Active bleeding visualized, No. (%)	18 (25.0)	44 (59.5)	<.001	5.23 (2.23 to 12.27)	13 (24.1)	32 (59.3)	<.001	4.59 (2.00 to 10.49)
Presumed source of bleeding on initial test, No. (%)	46 (63.9)	55 (74.3)	.21	1.85 (0.81 to 4.20)	34 (63.0)	41 (75.9)	.21	1.86 (0.81 to 4.20)
Any invasive procedures, No. (%)	70 (97.2)	36 (48.7)	<.001	0.01 (0.001 to 0.08)	52 (96.3)	26 (48.2)	<.001	0.04 (0.01 to 0.16)
Invasive procedures, mean (SD)	1.18 (0.48)	0.59 (0.77)	<.001	−0.54 (−0.77 to −0.31)	1.11 (0.42)	0.63 (0.85)	<.001	−0.48 (−0.74 to −0.23)
Total diagnostic procedures, mean (SD)	1.30 (0.57)	1.62 (0.86)	.01	0.34 (0.085 to 0.59)	1.28 (0.56)	1.67 (0.95)	.01	0.39 (0.09 to 0.69)
PPE use, mean (SD)	4.78 (1.90)	3.38 (3.10)	.001	−1.23 (−2.12 to −0.33)	4.52 (1.63)	3.52 (3.41)	.06	−1 (−2.02 to 0.02)
Safety								
Change in hemoglobin, mean (SD), g/dL	3.68 (2.26)	3.72 (1.90)	.92	−0.25 (−0.97 to 0.47)	3.72 (2.06)	3.44 (1.76)	.45	−0.28 (−1.01 to 0.45)
Units pRBC transfused, mean (SD)	2.28 (2.48)	2.57 (3.23)	.55	−0.139 (−1.09 to 0.81)	2.56 (2.52)	2.50 (3.53)	.93	−0.06 (−1.23 to 1.12)
Rebleeding, No. (%)	10 (13.9)	12 (16.2)	.82	1.51 (0.53 to 4.23)	9 (16.7)	11 (20.4)	.81	1.28 (0.48 to 3.39)
Readmission for bleeding, No. (%)	7 (9.7)	10 (13.5)	.61	1.59 (0.48 to 5.24)	7 (13.0)	9 (16.7)	.797	1.34 (0.46 to 3.91)
In hospital mortality, No. (%)	2 (2.8)	3 (4.1)	>.99	1.57 (0.20 to 12.12)	2 (3.7)	2 (3.7)	>.99	1 (0.14 to 7.37)

Abbreviations: OR, odds ratio; PPE, personal protective equipment; pRBC, packed red blood cell; SOC, standard of care.

SI conversion: To convert hemoglobin to grams per liter, multiply by 10.

^a^ The *P* values for active bleeding and presumed source of bleeding were analyzed using the Fisher exact test (categorical variables). The other *P* values were analyzed using the *t* test (continuous variables).

^b^ Adjusted for sex; age; race; Glasgow-Blatchford score; use of nonsteroidal anti-inflammatory drugs, aspirin, warfarin, and direct oral anticoagulants; no anticoagulation; cirrhosis; kidney failure; and presenting symptoms.

**Table 3.** Number of Procedures in Each Treatment Group

Procedures	COVID-19, No. (%) (n = 74)	SOC, No. (%) (n = 72)
First diagnostic procedure		
EGD	0	60 (83.3)
Colonoscopy	0	9 (12.5)
VCE	74 (100)	3 (4.2)
Total procedures		
EGD	21 (28.4)	61 (84.7)
Colonoscopy	9 (12.2)	21 (29.2)
VCE	74 (100)	6 (8.3)
Enteroscopy	8 (10.8)	1 (1.4)
CT angiogram	0	2 (2.8)
Surgery	1 (1.4)	2 (2.8)

Abbreviations: CT, computed tomography; EGD, esophagogastroduodenoscopy; SOC, standard of care; VCE, video capsule endoscopy.

### Outcomes

For the primary outcome, active bleeding was detected in 44 patients (59.5%) in the COVID-19 group compared with 18 (25.0%) in the SOC group (OR, 5.23; 95% CI, 2.23-12.27) (Table 2). When the patient population was matched by propensity scoring, the OR decreased slightly to 4.75 (95% CI, 2.02-11.14). A presumed bleeding source was identified using the first diagnostic modality in 55 patients (74.3%) in the COVID-19 group compared with 46 patients (63.9%) in the SOC group (OR, 1.85; 95% CI, 0.81-4.20).

In the overall population, only 36 (48.7%) patients in the COVID-19 group underwent any invasive procedures compared with 70 (97.2%) in the SOC group (adjusted OR, 0.01; 95% CI, 0.001 to 0.08). The mean (SD) number of invasive procedures was 0.59 (0.77) per patient in the COVID-19 group compared with 1.18 (0.48) per patient in the SOC group (adjusted difference, −0.54; 95% CI, −0.77 to −0.31). The mean (SD) number of diagnostic procedures was 1.62 (0.86) per patient in the COVID-19 group compared with 1.30 (0.57) per patient in the SOC group (adjusted difference, 0.34; 95% CI, 0.085 to 0.59). There was no statistically significant difference in the rates of rebleeding, inpatient mortality, hemoglobin level decrease, or units of packed red blood cell transfusions in the overall population or the matched population (Table 2). There was no mortality attributed to GI bleeding in either group.

The matched population, but not the overall population, showed a significant difference in localization of bleeding (Table 4). When analyzed separately against other localizations, the COVID-19 group showed higher rates of bleeding in the midgut compared with the SOC group (11 of 74 [15%; 95% CI, 7.7%-25.0%] vs 2 of 72 [3%; 95% CI, 0.3%-9.7%]). Distribution of the type of lesion identified also differed (*P* < .001 in overall; *P* < .001 in matched). Angioectasias were the most common finding (18 [24%]) in the COVID-19 group; peptic ulcer disease was most common in the SOC group (22 [31%]) (Table 4).

**Table 4.** Bleeding Localization and Lesion Type

Variable	Overall population			Matched population		
	COVID-19 (n = 74)	SOC (n = 72)	*P* value	COVID-19 (n = 54)	SOC (n = 54)	*P* value
Bleeding localization, No. (%)						
Upper GI	35 (47)	38 (53)	.08	24 (44)	31 (57)	.03
Midgut	11 (15)	2 (3)		10 (18)	2 (4)	
Lower GI	11 (15)	11 (15)		9 (17)	4 (7)	
Type of lesion, No. (%)						
Peptic ulcer	15 (20)	22 (31)	<.001	10 (19)	18 (33)	<.001
Angioectasia	18 (24)	5 (7)		15 (28)	5 (9)	
Blood without discrete lesion	8 (11)	2 (3)		5 (9)	1 (2)	
Dieulafoy	3 (4)	1 (1)		2 (4)	1 (2)	
Varices	1 (1)	4 (5)		1 (2)	3 (6)	
Mallory-Weiss tear	0	2 (3)		0	2 (4)	
Diverticulosis	0	5 (7)		0	3 (6)	
Portal hypertension	4 (5)	1 (1)		4 (7)	1 (2)	
Other	9 (12)	8 (11)		6 (11)	16 (29)	

Abbreviations: GI, gastrointestinal; SOC, standard of care.

Based on assumptions described earlier, we estimated 250 personnel encounters and sets of PPEs used for endoscopic evaluation of patients in the COVID-19 group (mean [SD], 3.38 [3.10] PPE/patient) compared with 344 personnel encounters and sets of PPEs in the SOC group (mean [SD], 4.78 [1.90] PPE/patient) (adjusted difference, −1.23; 95% CI, −2.12 to −0.33) (Table 2). This calculation was associated with an estimated 30% reduction in the overall use of PPE.

## Discussion

In this cohort study, we noted that the initial use of VCE as an alternative to the traditional, more invasive diagnostic evaluation of GI bleeding with EGD and/or colonoscopy appeared to be safe during the COVID-19 pandemic. When used early, VCE as the first strategy was associated with improved localization of active bleeding and reduced the number of urgent invasive endoscopic evaluations without increasing the risk of complications. In addition, VCE as the first strategy was associated with reduced staff use, and thereby reduced risk of exposure to endoscopic aerosols and conserved PPE.

Using VCE as a triaging tool before endoscopic evaluation is a new frontier in the management of GI bleeding described in recent randomized clinical trials.^9,12^ The data presented herein are consistent with an earlier observation in nonhematemesis GI bleeding that showed improved localization of the anatomic source of bleeding in 64% of patients in the VCE group compared with 31% in the SOC group.^9^ Previous data have shown that patients can be risk stratified using a combination of VCE data and clinical parameters and safely discharged if there is no evidence of active, ongoing bleeding. Therefore, in this setting, VCE serves as a triaging tool for endoscopic localization and to help determine the need for further invasive endoscopic intervention, because clinical history alone can be imprecise in localization and VCE provides additional clinical information beyond nasogastric tube aspiration^13^ and the Glasgow-Blatchford or Rockall scoring systems.^14,15^ Although current guidelines recommend early endoscopy within 24 hours for acute GI bleeding, studies have suggested that some invasive procedures can be safely deferred in the acute setting in carefully selected stable patients. A recent meta-analysis by Tsay and colleagues^16^ suggested that colonoscopy within 24 hours did not decrease the risk of rebleeding or mortality in patients hospitalized with acute lower GI bleeding. A similar study by Aziz and colleagues^17^ compared EGD within 24 hours of presentation to EGD after 24 hours of presentation and found no significant difference in mortality, recurrent bleeding, or length of stay. Because previous studies suggest that the yield of VCE is higher if the procedure is performed earlier,^10,18^ our findings presented herein suggest that earlier use of VCE along with clinical parameters may aid in risk stratifying patients who might require more urgent intervention vs those who can likely await further outpatient evaluation.

In our study, although there was a significant difference in the identification of active bleeding or stigmata of recent bleeding in the COVID-19 group, there was no statistically significant difference in attribution to a presumptive source of bleeding between the 2 groups, although the rate was still numerically higher in the COVID-19 group compared with the SOC group. A presumptive source of bleeding, common with the traditional approach to evaluation of GI bleeding, should be viewed with appropriate clinical skepticism to avoid premature closure. Bleeding attributed to findings such as small, clean-based ulcers, esophagitis,^19^ or diverticular disease^20,21^ without stigmata of active bleeding should be viewed within the proper clinical context to avoid easily missed diagnoses, such as Cameron lesions,^22^ Dieulafoy lesions,^23^ or small bowel lesions.^18^ Given the increased rates of detection of active bleeding or stigmata of bleeding and hence potentially better localization with early deployment of VCE as noted in this study and others, VCE potentially provides additional benefits via a more complete view of the GI tract.^18^

As noted, VCE use has additional advantages over SOC. Endoscopy during the COVID-19 pandemic provides additional challenges for infection control, limited PPE availability,^24^ and need for staff redeployment.^25^ Traditional endoscopic procedures involve multiple personnel, including the endoscopist, the endoscopy nurse, technicians, anesthesia staff, and trainees. Additional staff help with the patient’s preoperative assessment, recovery, processing of equipment, room turnover, and cleaning.^26,27^ To mitigate infectious risks, some centers, including both study sites, adopted policies such as canceling nonurgent procedures,^28^ prophylactic intubation of COVID-19–positive patients undergoing endoscopy, limited trainee participation, and reduced number of staff involved. However, prophylactic intubation has been associated with increased rates of bacterial pneumonia^29^ and prolonged intubation, and limiting trainee participation can reduce learning opportunities.^30^

In contrast, VCE is particularly suitable for triaging and diagnostic evaluation of patients with suspected or established COVID-19 because it requires no sedation or endotracheal intubation and minimal patient contact is needed to help set up equipment without contact with the oropharyngeal or gastrointestinal mucosa. In addition, the video capsule is disposable, and the recording equipment can be easily covered and cleaned after use. The advent of the real-time viewers built into the recording equipment allows quick real-time evaluation^31^ of the stomach and upper GI tract within minutes to help triage patients who would benefit from further therapeutic or additional diagnostic evaluations. Use of VCE in the COVID-19 setting is not a substitute for appropriate less-urgent screening or surveillance programs for cancer or other diagnostic evaluations, which can safely be deferred for conventional endoscopy. However, VCE is a useful tool to detect active bleeding or stigmata of recent hemorrhage and, combined with other clinical data, can be used in deciding the need for site-directed endoscopy.

Video capsule endoscopy has the disadvantage of being a passive device that cannot be manipulated after ingestion, but this is a small disadvantage because the goal is not necessarily to reach an exact diagnosis, but rather to risk stratify and triage patients who may benefit from additional therapeutic interventions. Furthermore, VCE is currently a purely diagnostic test that does not offer therapeutic options—a disadvantage compared with EGD and colonoscopy. However, it has been shown that many patients do not need therapeutic interventions and can be spared from these invasive procedures, at least in the acute setting.^12^ Our findings herein are consistent with those reported in a randomized clinical trial in which active bleeding was detected more than twice as frequently by VCE than SOC and 80% of patients^12^ reporting hematemesis could safely be discharged for subsequent outpatient evaluation.

Although a full cost analysis was outside the scope of this study, it is reasonable to estimate that there would be a potential associated reduction in expenses by decreased use of procedures and PPE. In a previous cost analysis of the use of early VCE in nonhematemesis GI bleeding, Jawaid and colleagues^32^ found no significant difference in total direct costs between patients who underwent early VCE compared with SOC. However, the costs were more efficiently distributed in the VCE group toward detection and treatment of bleeding, whereas in the SOC group, costs were incurred without obtaining a definitive diagnosis. Furthermore, Jawaid and colleagues projected that a more aggressive, albeit safe, discharge strategy could result in a 50% reduction in length of stay in the VCE vs SOC group (0.88 vs 1.63 days), with an associated decrease in direct costs attributed to less patient time spent in the hospital and less time occupying a bed. These potential savings are likely to be expanded in the COVID-19 era, owing to the costs of PPE and additional costs incurred for added hospital stays due to testing requirements (COVID-19 testing) before many endoscopic procedures requiring sedation.

### Limitations

This study had several limitations. First, by its design, as necessitated by limitations during the COVID-19 pandemic, this was a retrospective study, which raises the potential for selection bias and lack of blinding. Herein, we have used propensity score matching to adjust for some of these differences. Larger, multicenter, prospective randomized clinical trials are necessary to generalize the results to other populations. Second, there was a disproportionate number of patients with hematochezia in the SOC group, which raises a question of bias. Despite this difference, a similar number of patients had lesions localized to the colon in both groups, most prominently in the proximal colon in the VCE group, arguing that not all right colonic bleeds are being identified by the SOC approach, either due to delay or the preparation for colonoscopy.

## Conclusions

The findings of this cohort study suggest that the initial use of VCE as an alternative to the traditional, more invasive diagnostic evaluation of GI bleeding with EGD and/or colonoscopy appeared to be a safe and useful alternative in the era of the COVID-19 pandemic. The use of VCE vs SOC was associated with a higher detection rate of stigmata of bleeding, reduced use of traditional endoscopic procedures requiring sedation, and reduced personnel and PPE use without a significant increase in transfusion requirements, rebleeding rates, or mortality.

## Notes

Supplement. **eTable 1.** Full List of Baseline Characteristics **eTable 2.** Match Variables in Matched Populations **eTable 3.** Secondary Analysis of Primary Outcome
