# Supplementary material for: Assessment of Video Capsule Endoscopy in the Management of Acute Gastrointestinal Bleeding During the COVID-19 Pandemic
Source: JAMA Netw Open. 2021 Jul 30;4(7):e2118796. doi: 10.1001/jamanetworkopen.2021.18796 (PMC8325069; doi:10.1001/jamanetworkopen.2021.18796)
Supplement: Supplement. — eTable 1. Full List of Baseline Characteristics eTable 2. Match Variables in Matched Populations eTable 3. Secondary Analysis of Primary Outcome [file jamanetwopen-e2118796-s001.pdf]

Supplementary Online Content

Hakimian S, Raines D, Reed G, et al. Assessment of video capsule endoscopy in the management of acute gastrointestinal bleeding during the COVID-19 pandemic. *JAMA Netw Open*. 2021;4(7):e2118796. doi:10.1001/jamanetworkopen.2021.18796

eTable 1. Full List of Baseline Characteristics

eTable 2. Match Variables in Matched Populations

eTable 3. Secondary Analysis of Primary Outcome

This supplementary material has been provided by the authors to give readers additional information about their work.

© 2021 Hakimian S et al. *JAMA Network Open.*

eTable 1. Full list of baseline characteristics		
	**Covid (n=74)**	**SOC (n=72)**
Age, mean (SD), y	64.9 (14.1)	61.3 (13.4)
**Sex**		
Male	45 (61)	47 (65)
Female	29 (39)	25 (35)
**Race/ethnicity**		
Caucasian	49 (66)	40 (56)
African American	12 (16)	16 (22)
Hispanic	4 (5)	6 (8)
Other	9 (12)	10 (14)
**Comorbidities**		
COPD	13 (18)	12 (17)
Congestive heart failure *	23 (31)	12 (17)
Coronary artery disease	27 (36)	24 (33)
Cirrhosis	13 (17)	17 (24)
End-stage renal disease	5 (7)	4 (6)
Severe Aortic stenosis	2 (3)	1 (1)
Diabetes	28 (38)	25 (35)
**Other conditions treated during same hospitalization**		
Acute respiratory failure	14 (19)	10 (14)
Acute renal failure	18 (24)	12 (17)
Sepsis	8 (11)	7 (10)
Pneumonia	8 (11)	5 (7)
Hypoxemia	23 (31)	22 (31)
**ASA class**		
1	1 (1)	1 (1)
2	11 (15)	7 (10)
3	51 (69)	48 (67)
4	11 (15)	16 (22)
**Medication use**		
NSAID use	9 (12)	14 (20)
Aspirin	33 (45)	27 (38)
Heparin	2 (3)	2 (3)
P2Y12 inhibitors	10 (14)	8 (11)
Warfarin	14 (19)	8 (11)
Direct oral anticoagulants	10 (14)	4 (6)
No anticoagulation	33 (45)	39 (54)

© 2021 Hakimian S et al. *JAMA Network Open.*

Baseline HGB (mg/dl), mean (SD)	10.5 (2.6)	11.3 (2.4)
INR>2	14 (19)	6 (9)
BUN/cr>20	48 (65)	44 (61)
Glasgow-Blatchford Score, mean (SD)	8.4 (2.4)	7.6 (2.9)
**Presenting symptom**		
Hematemesis	8 (11)	11 (15)
Melena	35 (47)	27 (38)
Hematochezia *	3 (4)	16 (22)
Anemia	28 (38)	18 (25)
Presenting with GI bleeding	51 (69)	53 (74)

Abbreviations: SOC, standard of care; COPD, chronic obstructive pulmonary disease, pRBC, packed red blood cell; NSAID, non-steroidal anti-inflammatory drugs; HGB, hemoglobin, INR, international normalized ratio.

© 2021 Hakimian S et al. *JAMA Network Open.*

eTable 2: Match Variables in matched population

		COVID		SOC		
variable		(N =54)		(N =54)		Std. Diff.
		Mean	±SD	Mean	±SD	
Age		61.46	±13.75	62.78	±12.70	-.099
GBS		8.04	±2.35	8.28	±2.43	-0.101
		N	(%)	N	(%)	
Gender						-0.077
Male		36	(66.7)	34	(63.0)	
Female		18	(33.3)	20	(37.0)	
Caucasian						-0.074
no		24	(44.4)	22	(40.7)	
yes		30	(55.6)	32	(59.3)	
African American						0.000
no		43	(79.6)	43	(79.6)	
yes		11	(20.4)	11	(20.4)	
Hispanic						0.000
no		50	(92.6)	50	(92.6)	
yes		4	(7.4)	4	(7.4)	
Other						0.103
no		45	(83.3)	47	(87.0)	
yes		9	(16.7)	7	(13.0)	
Hematemesis						0.053
no		46	(85.2)	47	(87.0)	
yes		8	(14.8)	7	(13.0)	
Melena						0.073
no		25	(46.3)	27	(50.0)	
yes		29	(53.7)	27	(50.0)	
Hematochezia						0.000
no		51	(94.4)	51	(94.4)	
yes		3	(5.6)	3	(5.6)	
Anemia						-0.122
no		40	(74.1)	37	(68.5)	
yes		14	(25.9)	17	(31.5)	

© 2021 Hakimian S et al. *JAMA Network Open.*

variable	COVID (N =54)		SOC (N =54)		Std. Diff.
Cirrhosis/Liver fail.					0.000
no	41	(75.9)	41	(75.9)	
yes	13	(24.1)	13	(24.1)	
ESRD					0.000
no	50	(92.6)	51	(94.4)	
yes	4	(7.4)	3	(5.6)	
NSAID use					-0.099
no	46	(85.2)	44	(81.5)	
yes	8	(14.8)	10	(18.5)	
Aspirin					-0.037
no	32	(59.3)	31	(57.4)	
yes	22	(40.7)	23	(42.6)	
P2Y12					0.056
no	47	(87.0)	48	(88.9)	
yes	7	(13.0)	6	(11.1)	
Warfarin					0.152
no	44	(81.5)	47	(87.0)	
yes	10	(18.5)	7	(13.0)	
Direct Oral anticoagulants					0.200
no	48	(88.9)	51	(94.4)	
yes	6	(11.1)	3	(5.6)	
Heparin					0.000
no	53	(98.1)	53	(98.1)	
yes	1	(1.9)	1	(1.9)	
No Anti-Coagulant					-0.037
no	28	(51.9)	27	(50.0)	
yes	26	(48.1)	27	(50.0)	

© 2021 Hakimian S et al. *JAMA Network Open.*

eTable 3- secondary analysis of primary outcome

Active Bleeding Visualized<br>Models	Odds Ratio (COVID vs SOC)	95% Confidence Interval
Full population, MV adjusted[1]	5.23	2.23, 12.27
Full population on common support, MV adjusted[1]	5.08	2.12, 12.16
Matched Population	4.59	2.00, 10.49
Matched Population, adjusted for imbalance[2]	4.75	2.02, 11.14

1: adjusted for gender, age, race, GBS, NSAID use, aspirin, Warfarin, direct oral anti-coagulants, no anti-coagulation, cirrhosis, ESRD, presenting symptoms
2: Warfarin, direct oral anti-coagulants

© 2021 Hakimian S et al. *JAMA Network Open.*
